# Pediatric heart transplantation: Current status and perspectives

**DOI:** 10.1016/j.jhlto.2025.100353

**Published:** 2025-07-21

**Authors:** Estela Azeka, Fabiane Hase, Yoshihiro Tomimoto

**Affiliations:** a Instituto do Coracao (InCor), Hospital das Clinicas HCFMUSP, Faculdade de Medicina, Universidade de Sao Paulo, Sao Paulo, Brazil; b Undergraduate School, Faculdade de Ciencias Médicas e da Saude-Pontificia Universidade Católica de São Paulo (FCMS PUC-SP), Sorocaba, SP, Brazil; c Undergraduate School, Keio University, Tokyo, Japan

**Keywords:** heart failure, pediatric heart transplantation, congenital heart disease, xenotransplantation, donor

## Abstract

**Background:**

Heart transplantation (HT) has been the treatment of choice for children with end-stage heart disease. Since the first transplant in 1967, almost 6 decades on, vast improvements have occurred, the number of transplants has increased over time, and post-transplant survival curve has progressively improved.

**Methods:**

The purpose of this review is to analyze the current status of pediatric HT, including epidemiology of heart failure, number of ventricular assist devices, number of transplants, and outcomes. The pretransplant, contemporary issues of children with heart failure, the waiting list for transplant, the role of multidisciplinary team, perspectives to overcome the storage of donors with xenotransplantation, donation after circulatory death, and ABO-incompatible transplantation are discussed.

**Results:**

The outcome of transplantation in children and the main post-transplant issues related to immunosuppression, transition, and adherence were addressed, as well as the use of telemedicine as an additional tool for surveillance.

**Conclusion:**

HT in pediatric population is an established treatment for end-stage heart disease patients. The survival has increased over time, and there is a promising improvement in the care of these patients in relation to quality of life based on advances in the current researches and research and innovations in the future, improving life expectancy.

## Background

Heart transplantation (HT) has been the treatment of choice for children with end-stage heart disease.

Since the first transplant in 1967,^1^ almost 6 decades on, vast improvements have occurred, the number of transplants has increased over the last decades, and post-transplant survival curve has progressively improved. The advances in the pretransplant, perioperative management, and long-term surveillance have contributed to achieving these findings. Despite these advances and immunological knowledge, immunosuppressive drugs cannot completely prevent rejection episodes and have adverse effects. In addition, clinical trials in the pediatric population are still scarce, both in patients with heart failure (HF) awaiting transplantation and after transplantation.

The purpose of this review is to analyze the current status of pediatric HT, including epidemiology of HF, number of ventricular assist devices, number of transplants, and outcomes. The pretransplant, contemporary issues of children with HF, the waiting list for transplant, the role of multidisciplinary team, outcomes of transplantation, perspectives to overcome the storage of donors with xenotransplantation, donation after circulatory death (DCD), and ABO-incompatible (ABOi) transplantation are addressed. The outcome of transplantation in children and the main post-transplant issues related to immunosuppression, transition, and adherence were discussed, as well as the use of telemedicine as an additional tool for surveillance.

## Epidemiology

HF is one of the important health care system issues. In 2016, there were 7,932 visits to emergency department related to HF in children and 5.3% required HT in the United States.^2^ A systematic literature review on the incidence and prevalence of HF in children and adolescents described for primary HF, the incidence ranged from 0.87/100,000 (UK and Ireland) to 7.4/100,000 (Taiwan).^3^ Recently, the prevalence of HF among adolescents and young adults in 2021 was 14.4 per 100,000 population, increased from 12.2 in 1990, respectively.^4^

The percentage of recipients supported on a ventricular assist device submitted to transplant increased from 13.5% during 2001-2009 to 25% during 2010-2018, according to the International Thoracic Organ Transplant Registry of International Society for Heart and Lung Transplantation (ISHLT).^5^

The Advanced Cardiac Therapies Improving Outcomes Network Ventricular Assist Device registry reported from April 2018 to June 2023, 1,430 devices were implanted in 1,220 pediatric patients (less than 18 years) across North America.^6^ From those patients who were discharged, 50% were readmitted for transplantation.^6^

Pediatric Heart Transplant Society database described 13,311 recipients listed and 9,823 transplants performed from 1993-2025^7^ worldwide.

Recently, the International Thoracic Organ Transplant Registry reported that 1-year post-transplant survival improved from 87% during 2000-2005 to 92% during 2012-2017^5^ in pediatric population less than 18 years of age.

Hayes et al reported from Pediatric Heart Transplant Society database the long-term survival in children following HT. From 1993-2010, 1,610 patients ≤10 years of age at time of transplant and with conditional graft survival >3 years and at ≥10 years were analyzed. Risk factors for graft loss after 3 years post-transplant were malignancy, rejection, cardiac allograft vasculopathy (CAV), age, congenital heart disease, female sex, and black race. They concluded that HT is an effective therapy with a growing number of long-term survivors.^8^

## Challenges in pretransplant

Pretransplant issues are mainly divided into managing patients on the waiting list (bridge to transplant) and addressing donor shortages.

### Indication criteria and current treatments for patients on the waiting list (bridge to transplant)

Although HT is the definitive treatment for patients with advanced HF whose symptoms continue to worsen despite receiving maximally optimized Guideline-Directed Medical Therapy and device therapy, new drugs for treatment of HF and development of strategies and technologies to decrease stroke in children with ventricular assist devices play an important role in the current era.^9^

Pediatric transplant candidates face unique challenges, requiring both medical and nonmedical interventions. Pharmacotherapy includes standard drugs such as angiotensin-converting enzyme inhibitors, angiotensin-receptor blockers, beta-blockers, mineralocorticoid receptor antagonists (spironolactone), angiotensin-receptor neprilysin inhibitor (sacubitril/valsartan), and ivabradine. In acute decompensated HF, intravenous inotropes (e.g., dobutamine or milrinone) and early mechanical circulatory support are considered. New drugs that have emerged in children with HF as well as the titration of medications are dapagliflozin (sodium-glucose cotransporter-2 inhibitors), mineralocorticoid receptor antagonists (finerenone), and vericiguat.^10^, ^11^, ^12^, ^13^

The indications for HT in children include cardiomyopathies (dilated, restrictive, and hypertrophy), myocarditis, and complex congenital heart disease, repaired or not (Table 1).^14^, ^15^ According to the last ISHLT registry report,^5^ there were regional differences in distribution of dilated cardiomyopathy, congenital heart disease, and retransplantation as indications for transplant. In Europe, more than 50% of pediatric recipients had HT for dilated cardiomyopathy and almost 25% recipients had congenital heart disease. In North America, dilated cardiomyopathy and congenital heart disease were equally prevalent diagnoses in recipients. In other regions of the world, dilated cardiomyopathy was the most prevalent diagnosis.^5^ The ISHLT stratified pediatric HF into 4 stages (stages A-D) in Table 2.^16^

**Table 1:** Main Indications for Heart Transplantation^14^

Growth failure secondary to heart failure
Need for mechanical circulatory support or intravenous inotropic
Malignant arrhythmia or survival of cardiac arrest unresponse to catheter ablation, or automatic implantable defibrillator
Progressive pulmonary hypertension
Unacceptably quality of life

**Table 2:** Heart Failure Staging in Children^16^

A. Patients with increased risk of developing HF, but who have normal cardiac function and no evidence of cardiac chamber volume overload. B. Patients with abnormal cardiac morphology or cardiac function, with no symptoms of HF, past or present. C. Patients with underlying structural or functional heart disease and past or current symptoms of HF. D. Patients with end-stage HF requiring continuous infusion of intropic agents, mechanical circulatory support, cardiac transplantation, or hospice care.

Abbreviation: HF, heart failure.

Furthermore, the complexity of recipient profile on the transplant waiting list has changed over time.^5^ Children with complex congenital heart disease, single ventricle physiology submitted to palliative surgeries, failing Fontan circulation, 1.5 ventricle repair for congenital heart disease with tricuspid regurgitation as well as ventricular dysfunction after repair have been increasing in the recent era; the cardiac transplant in this population has allowed an improvement in the quality of life. However, pretransplant management and the survival following transplantation have been still a challenge.^17^, ^18^, ^19^, ^20^

Other key factors in evaluating the patient on the list are pulmonary vascular resistance, low cardiac output, and clinical deterioration signs (e.g., inotrope dependence, recurrent HF admissions) and worsening of Interagency Registry for Mechanically Assisted Circulatory Support classification. Exclusion criteria include active cancer, irreversible organ failure, severe infections, and inadequate social support. In addition, for infants with neuromuscular disorders or chromosomal abnormalities, individualized assessments are required, considering post-transplant complications and impacts on quality of life. Intellectual disability alone is not recommended as an exclusion criterion for transplant. A multidisciplinary approach is emphasized for infants with developmental delays or neuromuscular diseases.^15^

The ISHLT guidelines for the care of heart transplant recipients^21^ have been updated and important topics have been addressed, such as frailty, nutritional issues, mechanical circulatory support as a bridge for HT as well as support for patients with high pulmonary hypertension and cancer that preclude HT. Multidisciplinary professionals as well as palliative care team are recommended to support the patient and family. Post-transplant nurse coordinators should be involved in coordinating the care of inpatient and outpatient HT recipients. Furthermore, physical therapists and occupational therapists are beneficial in the post-transplant care of HT recipients, to promote early mobilization and rehabilitation. Adolescents also require education on treatment adherence and post-transplant life.

Another issue is maintaining normal growth on waiting list. Many children need nutritional support via nasogastric feeding, gastrostomy, or total parenteral nutrition to prevent malnutrition.

The prophylaxis of infectious episodes is an important topic and includes completing vaccines (e.g., influenza, pneumococcal, hepatitis B) and screening for cytomegalovirus, Epstein-Barr virus, and toxoplasmosis.^21^

### Addressing donor shortage

Waitlist mortality rates vary from 17% to 30% by country and institution across the globe. In Japan, it is described to be 30%, Australia 22%, Eurotransplant 18%, and United States 17%.^22^

Furthermore, there are worldwide disparities in pediatric donor heart usage. It has been described that around 40% of offered organs are not utilized. The nonutilization of donors is multifactorial: pediatric donor heart assessment and subsequent acceptance guidelines; disparate regulatory requirements; regional differences in prioritization schemes, health care system issues, and availability of bridging devices. Nguyen et al^23^ reported that of 72,460 pediatric/young adult heart donations, 41,065 (56.7%) were transplanted, and 31,395 (43.3%) were unutilized. The average annual number of discarded hearts in different periods (1987-2000), (2000-2010), and (2010-2022) was 791 (42.8%), 1,035 (46.3%), and 843 (41.2%), respectively.

Technological solutions to donor shortages focus on xenotransplantation and advances in regenerative medicine, both of which have the potential to provide alternatives to human HT in the future.

#### Alternatives to transplantation

##### Xenotransplantation

Bailey et al^24^ reported the first transplantation of a baboon heart into a newborn (Baby Fae) with hypoplastic left heart syndrome and survived 20 days. The cardiac graft showed traces of cell-mediated rejection and a progressive, potentially avoidable humoral response. A successful modified pig heart into a human recipient was performed in 2022, and has survived for 2 months.^25^ A second case was reported in 2023 and survived for 40 days. In these cases, immune rejection and viral infections were major challenges.^26^

In the pediatric population, xenotransplantation models to understand the limitations in this age group are being carried out. Preliminary results indicate that pulmonary edema and pleural effusions in the setting of systemic inflammation preclude clinical progression. Further studies are necessary for survival.

Expanded access within pediatric xenotransplantation, informed consent for a pediatric patient, biosurveillance, and protection of certain bystanders are some ethical issues that need to be considered when preparing pediatric application for xenotransplantation.^27^

Advances in gene-editing technologies have reduced porcine antigenicity and enhanced immunomodulation; however, further improvements are required in immune rejection management and immunosuppressive therapies.

Xenotransplantation could also help to solve the issue of size matching, especially in pediatric patients, by providing appropriately sized donor hearts, offering significant potential for this population.^27^, ^28^, ^29^, ^30^

##### Regenerative medicine using pluripotent stem cells

Cardiomyocytes derived from human pluripotent stem cells, including human embryonic stem cells and induced pluripotent stem cells, are being studied for heart regeneration therapies. Major challenges are improving differentiation techniques, reducing tumorigenic risks, developing large-scale cell culture systems, and addressing post-transplant arrhythmias and rejection. Continued preclinical studies and technological improvements are necessary for the safe and effective clinical application of cardiac regenerative medicine.^31^

#### Use of DCD donors

In addition to traditional organ donation from brain-dead donors, DCD for HT is becoming more widespread, due to advances in organ preservation techniques. The TransMedics Organ Care System is a leading normothermic ex-situ perfusion system.

However, the TransMedics Organ Care System is indicated for donors weighing ≥50 kg, making it suitable mainly for adults and large pediatric patients. Recently, an early single-center experience with an ex vivo organ care system in pediatric HT was reported with a median follow-up period of 11.9 months and no deaths, suggesting that ex vivo perfusion is a valuable technique in pediatric HT.^32^, ^33^

Transplantation of hearts donated after circulatory determination of death has been utilized to increase the pool of pediatric donor allografts. The original approach included direct procurement and perfusion in the recipient, and recently ex-situ normothermic machine perfusion (NMP). However, NMP is not available to the smallest pediatric patients. An alternative method, normothermic regional perfusion, can overcome the limitations of NMP. Normothermic regional perfusion is a thoracoabdominal dynamic in-situ organ assessment method and is instituted following declaration after determination of circulatory death. In-situ tissue perfusion is established in the transplantable organs using a bypass circuit. Utilization of this technology can expand the donor pool for pediatric recipients.^34^, ^35^

Future efforts should focus on accumulating clinical experience, strengthening health care infrastructure, and addressing the high costs of perfusion systems. Ethical and legal issues related to restoring circulation after cardiac arrest also remain to be addressed.

#### ABO-incompatible donors

Improvements in donor-matching systems are essential. ABOi transplantation provides a significant solution to donor shortages, especially in pediatrics.

Between 2010 and 2017, the proportion of ABOi heart transplants in children under 2 years of age increased from 15% to over 30%, reflecting a growing acceptance of ABOi transplantation. This trend was influenced by the 2016 implementation of revised United Network for Organ Sharing (UNOS) guidelines, which allowed broader use of ABOi organs in infants. According to a study from 2010-2018 using the Pediatric Heart Transplant Society database, post-transplant outcomes of 1,288 infant heart transplant recipients (<2 years) were compared between ABOi and ABO-compatible (ABOc) transplants; the 1-year survival rate for the ABOi group (76.7%) was similar to that of the ABOc group (80.7%), and the 2-year survival rates were also comparable (70.5% vs 73.4%, respectively). Additionally, the incidence of acute rejection was similar between the 2 groups (8.46% for ABOi vs 7.86% for ABOc).^36^

Thus, ABOi transplantation offers a valuable solution for addressing donor shortages in infants and increases the donor pool. Some B or AB donor hearts that would otherwise have been discarded can be used and O blood type recipients can access a larger pool. Future efforts should focus on longer-term outcome studies and improvements in immunosuppressive therapies.^37^, ^38^

#### HLA (Human Leukocyte Antigen) desensitization

Sensitization is associated with prolonged waiting times, higher mortality on the waiting list, or ineligibility for transplantation.　Therefore, desensitization therapy is crucial for successful transplantation. The current standard desensitization treatments include mechanical antibody removal (plasmapheresis or plasma exchange) and antibody suppression [intravenous immunoglobulin (IVIG), rituximab, and bortezomib].^5^, ^21^ Moreover, emerging therapies are also under investigation. Promising novel agents include the IgG-degrading enzyme imlifidase, immunoadsorption, CTLA4-Ig (belatacept), and anti-IL-6 receptor antibodies such as tocilizumab. Desensitization plays a pivotal role in expanding transplant opportunities for sensitized patients, and novel agents may further improve outcomes in HT.

HLA desensitization is a potential strategy for transplant across a positive crossmatch.^39^ Pediatric HT across a positive crossmatch in the first year results from the CTOTC-04 multi-institutional study showed that freedom from antibody-mediated and cellular rejection was lower in the crossmatch positive group and/or in the presence of donor-specific antibodies (DSAs). Follow-up will determine if acceptable outcomes can be achieved long-term.

Daratumumab was reported to be a valuable tool for desensitization and antibody-mediated rejection (AMR) treatment; however, additional studies are needed to determine long-term outcomes.^40^

## Post-transplant challenges

### Survival

The last ISHLT registry shows Kaplan-Meier survival curves of 1-year survival, stratified by recipient age <1 year, 1 to 10 years, and 11 to 17 years, were 89%, 92% and 94%, respectively. Five-year conditional on surviving to 1 year was 91.2%, 92.3%, and 88%, respectively.^5^

The main complications after transplantation and cause of death described are infection, CAV, kidney injury, and post-transplant lymphoproliferative disorders.^5^, ^6^

### Immunosuppressive therapy

#### Induction protocols

Induction therapy with rabbit antithymocyte globulin or interleukin-2 receptor antagonists has been increased over time to prevent rejection in the perioperative period. The ISHLT registry reported that most patients have received this therapy.^5^

A multi-institutional study described the impact of induction therapy and outcomes after stratifying patients by diagnosis and risk and concluded that induction therapy was associated with decreased rejection, but it was not found to directly influence survival on multivariable analysis.^41^

#### Maintenance immunosuppressive therapies

Immunosuppressive therapies have improved over time, since the use of calcineurin inhibitor (cyclosporine) and antiproliferative agents as a second maintenance drug, such as azathioprine, which allowed improvement in survival curve. Nowadays, the ISHLT registry reported that tacrolimus (calcineurin inhibitor) and mycophenolate (antiproliferative agent) are the most common maintenance immunosuppressive drugs used after transplantation.^5^, ^21^

Steroids were one of the mainstays of immunosuppressive therapies for solid organ transplantation. However, their side effects were significant, including Cushingoid facies, obesity, hyperlipidemia, and severe growth retardation. Harmful effects of long-term steroid use can be avoided with the steroid-avoiding protocol while still achieving satisfactory immunosuppression, especially in pediatric population.^42^

There are differences between traditional and contemporary immunosuppressive agents. A comparison of traditional (cyclosporine, azathioprine, and steroid with no induction) vs contemporary (tacrolimus, mycophenolate mofetil, and induction) immunosuppressive regimens in pediatric heart recipients reported that a contemporary immunosuppression regimen was associated with less rejection in the first year, with no difference in the risk of infection but with risk of anemia and neutropenia requiring treatment. Long-term follow-up is needed to evaluate the impact of this regimen on survival.^43^

Newer agents, such as mammalian target of rapamycin inhibitors, are replacing traditional regimens in an effort to improve outcomes while decreasing immunosuppression-related complications.^44^ Tocilizumab is one of the new drugs that blocks the binding of interleukin 6 and may be effective in HT. Despite changes in immunosuppression protocols, other post-transplant protocols centers have remained unchanged, due to the need for long-term data on efficacy and safety. Changes in immunosuppressive regimens carry varying risks for post-transplanted patients. Variations in regimens across different centers might influence outcomes in pediatric HT.^44^

Rejection may be prevented with the evolution of immunosuppressants and newer strategies. There is a need for the development of newer immunosuppressant agents targeting different aspects of alloimmunity.

### Rejection

Pediatric HT has advanced considerably, improving survival rates and recipients’ quality of life. However, graft failure, graft rejection, CAV, and the need for retransplantation remain major challenges, which require innovative diagnostic strategies and close monitoring.^5^, ^21^ Therefore, time of detection of rejection is significant for the preservation of graft function. New noninvasive methods for detection of rejection, such as biomarkers, have been developed. Donor-derived cell-free DNA is one of the emerging tools that have been used but not accessed in all centers worldwide.^45^

Graft rejection is classified into AMR and acute cellular rejection. Recipients who develop AMR have worse cardiac vasculopathy outcomes than patients who have no rejection or develop acute cellular rejection.^46^ AMR has been associated with a higher risk of cardiovascular (CV) event, being caused by DSAs that result in vascular injury.

The time course of acute cellular rejection is usually in the first year of transplant, the induction therapy has increased over time, and new immunosuppressive regimens have improved the survival.^5^ Late rejection is associated with poor survival, especially when associated with hemodynamic compromise. Absence of late rejection episodes is associated with very low risk of death during medium-term follow-up after pediatric HT. Determining the risk factors for late rejection will help to identify a cohort of patients who may benefit from enhanced rejection surveillance and treatment. The outcomes with hemodynamically significant rejection have been worse than other types and grades of rejection.^47^

Webber et al reported from first results from the CTOTC-09 multi-institutional study that persistent first-year DSA was a risk factor for 3-year abnormal graft function.^48^

### Retransplantation

Pediatric heart retransplantation accounted for approximately 6% of all pediatric heart transplants. In the current era, survival after retransplantation shows promising results, which has been an option for those with advanced coronary allograft vasculopathy.^49^, ^50^

### Transition to adult care

Fortunately, the survival curve for children has improved and is making it possible for them to reach adulthood. Adherence post-transplant is crucial, especially during adolescent to young adult period. Nonadherence in this period has contributed to post-transplant mortality.^51^

Transition to adult care requires a multidisciplinary team that includes a pediatric physician, an adult provider, a social worker, a psychologist, a nurse, and a physiotherapist. TRANSIT (Pediatric heart transplantation: Transitioning to adult care feasibility of a pilot randomized controlled trial) was conducted, focused on increasing heart transplant knowledge, self-care, self-advocacy skills, and enhancing social support to improve outcomes (adherence to immunosuppressant, adverse events) for young emerging adults who underwent HT as children and transferred to adult care. They concluded in this pilot study that feasibility was demonstrated; however, the transition interventions did not improve the outcome.^52^ ISHLT is currently working on promoting guidelines so that the transition occurs effectively and with quality.

## Management using telemedicine

Effective post-transplant care requires continuous monitoring, early detection of complications, and immunosuppressive strategies. Therefore, telemedicine has become a post-transplant management critical tool, allowing access to specialized care while reducing risks.

Telehealth has been used to monitor heart transplant patients and has been identified as a viable modality for health care delivery. It shows promising applications in continuity of follow-up, medication titration, and patient counseling.^53^

Lower exercise capacities have been observed in pediatric heart transplant patients.^54^ Considering this, remote monitoring has facilitated the delivery of exercise and nutrition interventions. For instance, a study designed a 12- to 16-week diet and exercise program delivered via live video conferencing to improve cardiovascular health. However, studies show that adherence-promoting interventions and innovative solutions using technology are needed to enhance effectiveness.

One major complication is the lack of appropriate technical support, which might be mitigated with proper funding.^55^ Additionally, involving family members in certain aspects of telehealth programs has proven beneficial, since parental psychological impact is a crucial factor in implementing new technology in a home setting. This is especially relevant for telehealth applications that require parental involvement, such as parental home echo acquisition.^56^, ^57^

Despite the promising applications of telemedicine services, current challenges include licensure and regulatory barriers as well as the high costs of establishing a successful infrastructure.^58^

## Conclusions

HT in pediatric population is an established treatment for end-stage heart disease patients. The survival has increased over time, and there is a promising improvement in the care of these patients in relation to quality of life based on advances in the current research and innovations in the future, improving life expectancy.

## Disclosure statement

Estela Azeka reports a relationship with Merck and Co Inc that includes consulting or advisory.
